# Role of Neutrophil Gelatinase-Associated Lipocalin in the Diagnosis and Early Treatment of Acute Kidney Injury in a Case Series of Patients with Acute Decompensated Heart Failure: A Case Series

**DOI:** 10.1155/2016/3708210

**Published:** 2016-02-28

**Authors:** Silvia Angeletti, Marta Fogolari, Davide Morolla, Federico Capone, Sebastiano Costantino, Silvia Spoto, Marina De Cesaris, Alessandra Lo Presti, Massimo Ciccozzi, Giordano Dicuonzo

**Affiliations:** ^1^Clinical Pathology and Microbiology Laboratory, University Hospital Campus Bio-Medico, Via Alvaro del Portillo 200, 00128 Rome, Italy; ^2^Centro Integrato di Ricerche (CIR), Laboratory of Clinical Pathology and Microbiology, University “Campus Bio-Medico”, Via Alvaro del Portillo 200, 00128 Rome, Italy; ^3^Internal Medicine Department, University Hospital Campus Bio-Medico, Via Alvaro del Portillo 200, 00128 Rome, Italy; ^4^Department of Infectious, Parasitic and Immunomediated Diseases, National Institute of Health, Viale Regina Elena 299, 00161 Rome, Italy

## Abstract

Patients with acute decompensated heart failure (ADHF) frequently develop worsening in renal function until Acute Kidney Injury (AKI). The use of kidney injury biomarkers could be useful in the early diagnosis of AKI. In the present study, the role of the neutrophil gelatinase-associated lipocalin (NGAL), compared to the standard creatinine, in ADHF patients, was analyzed to evaluate if an early treatment could affect the outcome. A case series of 24 ADHF patients was enrolled and patients randomly divided in two groups (Group A and Group B). In Group A, NGAL, creatinine, and eGFR were measured, while in Group B, creatinine and eGFR alone were measured. NGAL was measured by turbidimetric immunoassay and creatinine using an enzymatic spectrophotometric method. In presence of AKI, creatinine increase and eGFR decrease were significantly lower in Group A than in Group B, whereas in absence of AKI the difference between the two groups was not significant. Hospitalization stay was significantly lower in Group A (receiving early treatment based on NGAL) than in Group B. In ADHF patients, plasma NGAL in combination with creatinine was superior to the standard creatinine in the diagnosis and early treatment of AKI with a better outcome and a decreased hospital stay.

## 1. Introduction

Acute Kidney Injury (AKI) is a clinical syndrome, characterized by a sudden decline in renal function, commonly affecting hospitalized patients [1]. In recent years, AKI incidence has been increasing especially in the emergency department (ED) settings [2, 3]. It has been estimated that an episode of AKI occurs, on average, in 5–10% of all hospitalized patients and in 40% of critically ill patients [4] and it is associated with increased risk of death [5]. Furthermore, AKI can increase the risk of infection, cause damage to other organs and systems (central nervous system, cardiovascular system), and raise the rate of rehospitalization. Even after the apparent resolution of an AKI episode, there is evidence of a significant increase in long-term risk of cardiovascular disease, or chronic kidney disease and overall mortality [2].

Patients affected by acute decompensated heart failure (ADHF) frequently develop worsening in renal function during hospitalization, configuring a complex pathological condition described as cardiorenal syndrome type 1. AKI occurs in 25–40% of patients affected by acute heart failure and represents its most frequent complication [3, 4].

Since clinical signs and symptoms of acute renal damage are not specific, it is difficult to promptly diagnose AKI at admission [5–8]. Currently, the diagnosis of AKI requires serial measurement of laboratory markers and it is based mainly on serum creatinine evaluation, as suggested by the Risk, Injury, Failure, Loss, and End-Stage Kidney Disease (RIFLE) criteria, the Acute Kidney Injury Network (AKIN) criteria, and the recent Kidney Disease Improving Global Outcomes (KDIGO) practice guidelines for AKI [9–11].

AKI, defined and classified according to the RIFLE criteria, significantly correlates with mortality; in fact increasing RIFLE classes are linearly correlated with increased mortality [12].

The need for repeated serum creatinine evaluations and monitoring of urinary output for several days after admission could result in a delay in appropriate therapy [5, 13].

The use of kidney injury biomarkers could be useful in the early diagnosis of AKI. Furthermore, it could allow to distinguish AKI from other conditions (volume responsive renal dysfunction or normal renal function) and contribute to the identification of patients with Subclinical AKI, characterized by renal damage in absence of serum creatinine increase [14, 15].

Consequently, earlier AKI patients stratification as well as a specific treatment could be reached [16, 17].

Recently, the neutrophil gelatinase-associated lipocalin (NGAL) has been described as one of the most promising biomarkers in the management of patients with acute kidney disease. NGAL is one of the earlier protein released from the kidney after ischemic or toxic damages [18].

Several studies suggested that, in patients with ADHF, NGAL is elevated and its rise precedes the plasma creatinine increase. Furthermore, NGAL represented an independent risk factor of worse prognosis in patients with acute heart failure [19, 20].

In a systematic review and meta-analysis of observational studies, plasma NGAL level appeared to have a diagnostic and prognostic value for AKI, independently from serum creatinine increase [15]. De Berardinis et al. reported that plasma NGAL, with a cut-off of 196 ng/mL, in combination with NT-proBNP or BNP may be useful to predict WRF in patients with acute decompensated heart failure (ADHF) [21].

Urinary NGAL, although has been demonstrated to be a good biomarker in AKI, was not specific for clinically significant worsening renal function prediction in patients with acute heart failure [22, 23].

If the accuracy of plasma and urinary NGAL, as markers of Acute Kidney Injury (AKI), is well demonstrated and shared by the scientific community, the effects of persistent alteration of the glomerular filtration rate (GFR) on plasma and urinary NGAL remained undefined for years [24].

A recent observational study of patients with chronic kidney disease showed that the levels of plasma NGAL increase progressively with the reduction in glomerular filtration rate (GFR) [25].

As a result of the new findings emerging in this field new criteria have been proposed for the diagnosis of AKI in patients with chronic kidney disease (CKD), including the increase of pNGAL > 50% compared to the reference value for the stage CKD, or raising the cut-off of pNGAL to 400 ng/mL [26].

The aim of the present study was to evaluate the role of plasma NGAL, compared to the standard creatinine, in the early diagnosis of Acute Kidney Injury in a case series of patients with acute heart failure, in order to assess how an early treatment can actually affect the outcome. As secondary aim urinary NGAL was compared to plasma NGAL to evaluate its role in AKI diagnosis in patients with heart failure.

## 2. Methods

### 2.1. Patients Selection and Study Design

A longitudinal, prospective, single-centre, randomized, single-blind study, between February 2015 and June 2015, was performed in the Department of Internal Medicine at the University Hospital Campus Bio-Medico of Rome. A case series of 24 patients with diagnosis of heart failure (ADHF, Acutely Decompensated Heart Failure) was designed.

The clinical diagnosis of heart failure has been supported by the dosage of the NT-proBNP at admission [27] and the structural heart disease assessed by transthoracic echocardiogram. Clinical evaluation and vital signs were assessed daily in all patients. At admission, patients were randomly divided into two study groups (Group A and Group B). In Group A, plasma NGAL, urinary NGAL and creatinine were measured in all patients at admission (*T*0), after 12 hours (*T*1), after 24 hours (*T* = 2), after 48 hours (*T* = 3), and after 72 hours (*T* = 4). In Group B, creatinine alone was measured in all patients at admission (*T*0), after 12 hours (*T*1), after 24 hours (*T* = 2), after 48 hours (*T* = 3), and after 72 hours (*T* = 4).

The glomerular filtration rate (eGFR) based on serum creatinine, using the CKD-EPI equation, has been evaluated in all patients.

The diagnosis of AKI was placed on the basis of the KDIGO criteria, currently in use (increased serum creatinine > 0.3 mg/dL in 48 hours or >1.5 times the baseline value in less than 7 days, or urinary volume < 0.5 mL/kg/h for at least 6 hours), and, in patients of Group B, using the threshold value of NGAL plasma levels of 200 ng/mL, which represents one of the most accurate cut-off in AKI diagnosis for ADHF patients (21). In patients with chronic kidney disease the diagnosis was based on the criteria mentioned above or after the detection of NGAL values increase > 50% from the baseline reference value used for staging CKD.

All patients with heart failure received the proper treatment in accordance with the current guidelines of the European Society of Cardiology (ESC) [28]. In both Group A and Group B, when AKI was diagnosed, diuretic therapy was modulated, nephrotoxic drugs were suspended and all measures of support to ensure the recovery of the optimal hemodynamic, electrolytic, and acid-base balance and to minimize the risk of complications were taken.

The following exclusion criteria in patients selection were adopted: patients with diagnosis or suspicious of localized infection, sepsis, or Systemic Inflammatory Response Syndrome (SIRS). Inclusion criteria adopted were patients affected by congestive heart failure.

Participants provided their written consent to participate in this study. The study was approved by the Ethic Committee of the University Hospital Campus Bio-Medico, and authors have complied with the World Medical Association Declaration of Helsinki.

### 2.2. Plasma NGAL, Urinary NGAL, and NT-proBNP Measurement

The quantitative determination of NGAL in urine (uNGAL) and plasma (pNGAL) was performed by the automated new Dimension Vista 1500 (Siemens, Healthcare Diagnostics Inc, Italy), using a particle enhanced turbidimetric immunoassay with the commercially available kit (The NGAL Test, Bio Porto Diagnostics, Denmark). The plasma creatinine was determined using an enzymatic spectrophotometric IDM traced method by the automated analyzer new Dimension Vista 1500 (Siemens, Healthcare Diagnostics Inc., Italy). Plasma NT-proBNP was measured by the chemiluminescence method using the automated analyzer new Dimension Vista 1500 (Siemens, Healthcare Diagnostics Inc., Italy).

### 2.3. Statistical Analysis

Normal distribution of pNGAL, uNGAL, and plasma creatinine was analyzed. The Mann-Whitney test for independent samples was used to compare the following:

- Plasma creatinine increase found in Group A and Group B patients with diagnosis of AKI.
- Plasma creatinine increase found in Group A and Group B patients without diagnosis of AKI.
- eGFR reduction in Group A and Group B patients with diagnosis of AKI.
- eGFR reduction in Group A and Group B patients without diagnosis of AKI.
- Days of hospital stay in Group A and Group B patients.

*p* value ≤ 0.05 was considered statistically significant.

The correlation of the paired pNGAL and uNGAL values in patients of Group A was assessed by the Spearman rank correlation coefficient (*ρ*) and *p* values ≤ 0.05 were considered significant.

Chi-square test was used to evaluate the different distribution, according to the RIFLE and AKIN staging of AKI severity, among patients of Group A (measured with NGAL and creatinine) and Group B (assessed by creatinine alone). *p* values ≤ 0.05 were considered significant.

Data were analyzed using the statistical package MedCalc 13.2.2.0 (Med-Calc Software, Ostend, Belgium).

## 3. Results

### 3.1. Patients and Controls Characteristics

The characteristics of the study population randomly divided in the two groups (A and B) are reported in Table 1. 13/24 (54.16%) patients with ADHF developed AKI during hospitalization; none of them required admission at the Intensive Care Unit (ICU) or died for comorbidities complications. CKD was present in 7/24 (29.16%) patients; 5/13 (38.46%) patients with CKD developed AKI. The duration of hospital stay was the same in both Group A and Group B.

### 3.2. pNGAL, uNGAL, and Plasma Creatinine Average Values (Mann-Whitney for Independent Samples)

Mean, median, 25th, and 75th percentiles of pNGAL, uNGAL, plasma creatinine, and e-GFR values in Group A and Group B patients measured at *T* = 0, *T* = 1, *T* = 2, *T* = 3, and *T* = 4 are summarized in Tables 2 and 3, respectively.

### 3.3. Comparison of the Increase in Serum Creatinine between Group A and Group B Patients (Mann-Whitney for Independent Samples)

**Figure 1:**
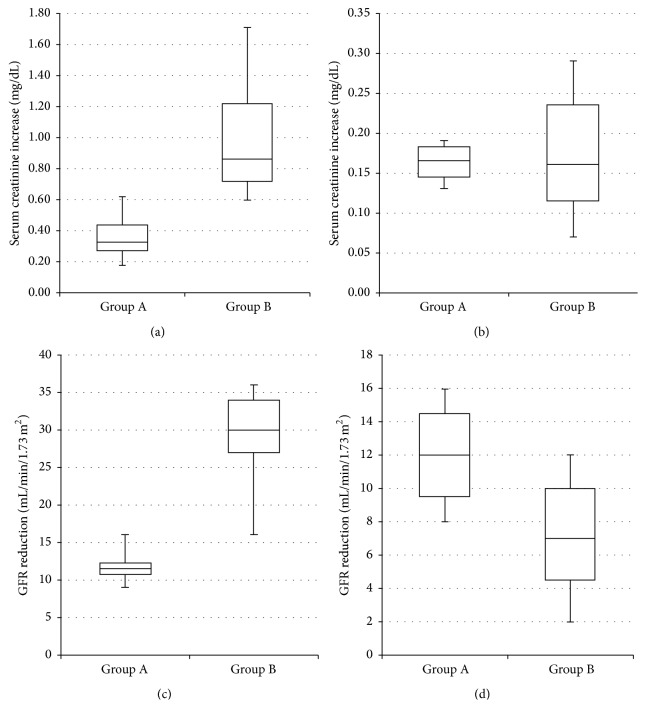
((a) and (b)) Comparison of the increase in serum creatinine between Group A and Group B patients (Mann-Whitney for independent samples). ((c) and (d)) Comparison of the decrease in eGFR between Group A and Group B patients (Mann-Whitney for independent samples).

Serum creatinine increase, in presence of AKI, was significantly lower (*p* = 0.007) in Group A patients (receiving early treatment based on NGAL) than Group B patients (evaluated only with creatinine), whereas in absence of AKI the difference between the two groups of patients was not significant (Figures 1(a) and 1(b)).

### 3.4. Comparison of the Decrease in eGFR between Group A and Group B Patients (Mann-Whitney for Independent Samples)

eGFR decrease, in presence of AKI, was significantly lower (*p* = 0.005) in Group A patients (receiving early treatment based on NGAL) than Group B patients (evaluated only with creatinine), whereas in absence of AKI the difference between the two groups of patients was not significant (Figures 1(c) and 1(d)).

### 3.5. Comparison of AKI Severity, Using RIFLE Criteria, between Group A and Group B Patients

**Figure 2:**
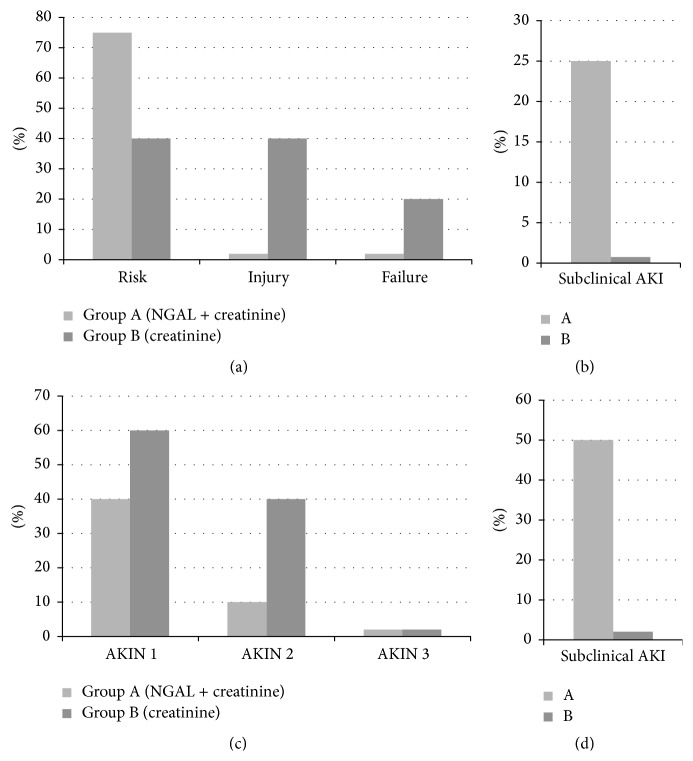
((a) and (b)) Comparison of AKI severity, using RIFLE criteria, between Group A and Group B patients. ((c) and (d)) Comparison of AKI severity, using AKIN criteria, between Group A and Group B patients.

Patients who developed AKI have been classified according to the RIFLE criteria. The distribution of these patients in various stages of severity was significantly different (*p* = 0.032) between Group A and Group B patients, as reported in Figure 2(a). Two patients who did not meet any of the RIFLE criteria, but had a significant elevation of plasma NGAL (above the cut-off of 200 ng/mL), have been placed in a separate group defined as Subclinical AKI (Figure 2(b)).

### 3.6. Comparison of AKI Severity, Using AKIN Criteria, between Group A and Group B Patients

Patients who developed AKI have been classified according to the AKIN criteria. The distribution of these patients in various stages of severity was significantly different (*p* = 0.036) between Group A and Group B patients, as reported in Figure 2(c).

Four patients who did not meet any of the AKIN criteria but had a significant elevation of plasma NGAL (above the cut-off of 200 ng/mL) have been placed in a separate group defined as Subclinical AKI (Figure 2(d)).

### 3.7. Comparison of Hospitalization Stay between Group A and Group B Patients Who Developed AKI (Mann-Whitney for Independent Samples)

**Figure 3:**
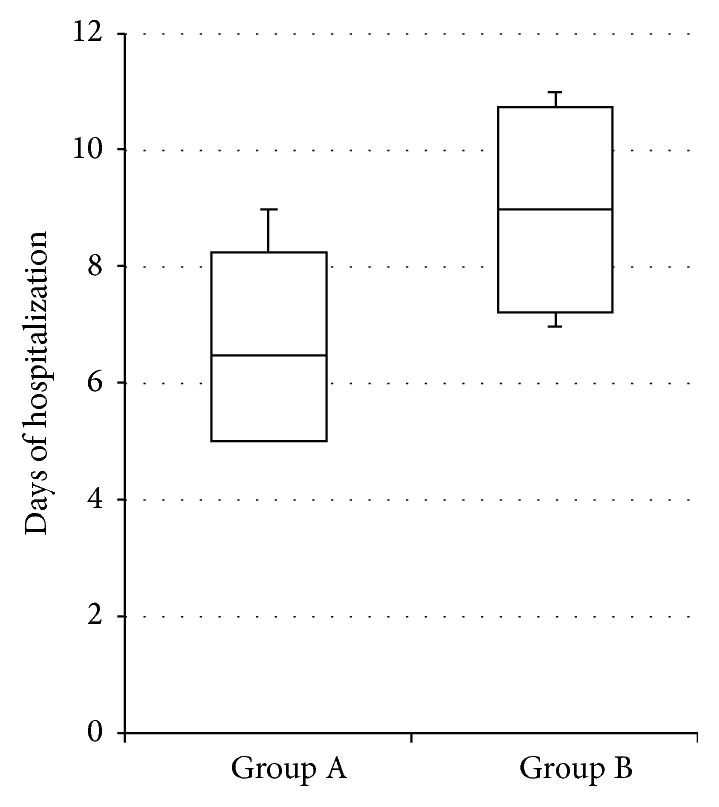
Comparison of hospitalization stay between Group A and Group B patients who developed AKI.

In presence of AKI, hospitalization stay, estimated in days, was significantly lower (*p* = 0.037) in Group A patients (receiving early treatment based on NGAL) than Group B patients (evaluated only with creatinine), as reported in Figure 3.

### 3.8. Rank-Correlation of uNGAL with Paired pNGAL Values in the Patients Population

**Figure 4:**
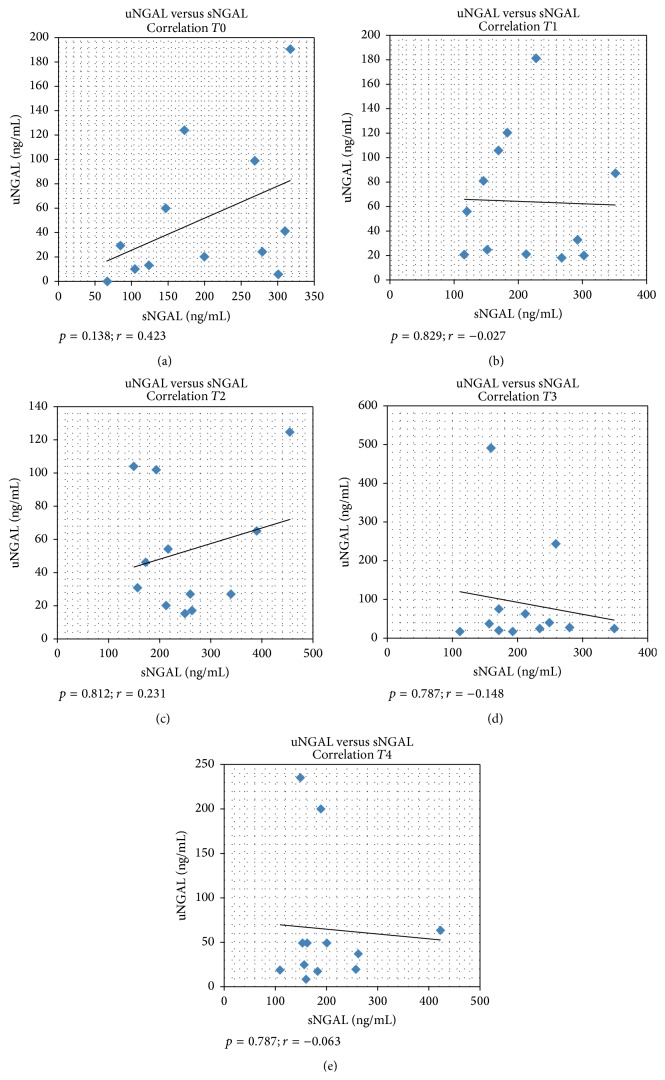
Rank-correlation of uNGAL with paired pNGAL values in the patients population. *p* = statistical significance;* r* = rank correlation coefficient.

At *T* = 0 (admission), *T* = 1 (12 hours after admission), *T* = 2 (24 hours after admission), *T* = 3 (48 hours after admission), and *T* = 4 (72 hours after admission), uNGAL values were not significantly correlated with pNGAL, as reported in Figure 4.

## 4. Discussion

Acute heart failure is the first cause of hospitalization in over 65-year-old patients. AKI develops in more than 25% of these patients and highly increases the risk of morbidity and mortality. Early recognition of AKI is the key to prevent short-term and long-term complications, to reduce the length of hospitalization and the costs of health assistance. In pediatric patients subjected to cardiopulmonary bypass, using NGAL, AKI identification was achieved within 2 hours from the renal damage [29].

NGAL has been described as a sensitive, specific, and early predictive biomarker for AKI and its diagnostic role in cardiorenal syndrome type 1 has been reported [30]. In this study, the role of NGAL in AKI diagnosis and subsequent treatment in patients with acute heart failure was evaluated with a cut-off value for plasma NGAL of 200 ng/mL.

Patients suffering from acute heart failure, who developed AKI during hospitalization, showed a minor downturn in the renal function when evaluated and treated using plasma NGAL, compared to the standard creatinine. These findings were supported by the analysis of both the increase in creatinine values and the reduction in the glomerular filtration rate (eGFR). AKI diagnosis time from admission was shorter in patients evaluated using plasma NGAL and creatinine, compared with those evaluated with creatinine alone.

The severity of Acute Kidney Injury (AKI), calculated according to the RIFLE and AKIN staging criteria, has proven to be lower in patients evaluated using plasma NGAL and creatinine than patients evaluated and treated using creatinine alone.

In addition, thanks to the use of NGAL, it was possible to identify those patients with Subclinical AKI which, using creatinine alone, would not have been identified. In these subjects, as in those with AKI, therapy was tailored to minimize the risk of renal complication (Figures 2(b) and 2(d)).

Subclinical AKI, as previously described, is characterized by renal damage in absence of serum creatinine increase, [14, 15]. Upon this, it cannot be identified with the criteria of diagnosis currently in use for kidney failure. This study suggested that the use of plasma NGAL in patients with ADHF could identify kidney damage before causing renal failure. Moreover the use of NGAL in AKI timely diagnosis could help clinicians act on nephrotoxic factors or drugs, reducing the risk of renal failure. Early treatment of kidney damage may help in preventing this frequent complication affecting patient's outcome.

The length of hospitalization of patients who developed Acute Kidney Injury was significantly lower in patients evaluated using plasma NGAL and creatinine than patients evaluated with creatinine alone as a consequence of the earlier begin of AKI treatment. This evidence could ensure not only a shorter hospital stay for the patient, but also a significant reduction in healthcare associated costs, which could justify the use of an additional marker in the diagnosis of acute kidney injury.

The correlation between urinary and plasmatic concentrations of NGAL was statistically not significant in every time analyzed (*T*0, *T*1, *T*2, *T*3, and *T*4), as already observed in other observational studies [23, 24].

Since NGAL has been demonstrated to rise in many different critical conditions such as sepsis or SIRS, an accurate identification of patient's disease is essential, in order to maximize its specificity and improve its role in clinical practice.

This study presents a main limitation: the small size of patients enrolled. This was due to the eligibility of patients for the study. In fact, all patients with localized infections, sepsis, or SIRS were excluded, in order to avoid false positive in the diagnosis of AKI as a consequence of the induction of NGAL by of proinflammatory cytokines in the neutrophils. A solution that would be able to circumvent this limit could be to measure also the monomer form of NGAL that is produced only by the kidney.

## 5. Conclusion

The use of plasma NGAL associated with creatinine in the diagnosis and early treatment of Acute Kidney Injury in patients affected by acute heart failure was superior in AKI severity prediction compared to the standard creatinine. A better outcome of patients with AKI as well as a decrease in the length of hospital stay was obtained. Results from this study showed the usefulness to include plasma NGAL measurement besides other routinely biomarkers, such as creatinine, in patients with cardiorenal syndrome to early identify AKI development.

## Figures and Tables

**Table 1:** Characteristics of the study population: a case series of 24 patients with acute decompensated heart failure (ADHF).

Patients	Group A	Group B
Number of patients	12	12
Mean age	81 ± 8	84 ± 8
Male	3	4
Female	9	8

AKI diagnosis	8	5
Absence AKI	4	7
Hospital stay (days)	7 ± 2	7 ± 2

CKD	4	3
COPD	1	4
Diabetes mellitus type 2	0	4
Chronic heart disease	12	12
Autoimmune vasculitis	0	0
Malignancy	1	3
Surgery	0	0

AKI: Acute Kidney Injury; CKD: chronic kidney disease; COPD: chronic obstructive pulmonary disease.

**Table 2:** Median, 25th, and 75th percentiles of plasma NGAl (pNGAL), urinary NGAL (uNGAL), plasma creatinine (pCrea), and e-GFR in Group A patients with and without AKI diagnosis.

Group A						
	AKI			Non-AKI		
	Median	25th^*∗*^	75th^*∗∗*^	Median	25th^*∗*^	75th^*∗∗*^
pNGAL (ng/mL)						
*T* = 0	274	162	305	125	86	159
*T* = 1	246	196	296	132	117	156
*T* = 2	261	230	365	164	153	194
*T* = 3	241	182	269	158	134	186
*T* = 4	196	172	260	130	130	159
uNGAL (ng/mL)						
*T* = 0	26	16	63	35	55	92
*T* = 1	29	20	102	68	38	93
*T* = 2	27	18	81	50	38	79
*T* = 3	27	23	55	50	26	89
*T* = 4	30	19	56	49	34	95
pCREA (mg/dL)						
*T* = 0	1.3	0.9	1.6	1.0	0.8	1.1
*T* = 1	1.3	1.1	1.5	1.0	0.8	1.0
*T* = 2	1.3	1.1	1.6	1.0	0.9	1.1
*T* = 3	1.2	1.1	1.6	1.0	0.8	1.0
*T* = 4	1.4	1.6	1.9	0.9	0.9	1.1
e-GFR (mL/min/1.73 m^2^)						
*T* = 0	45	36	54	56	49	72
*T* = 1	44	35	47	57	51	72
*T* = 2	45	34	48	51	48	66
*T* = 3	43	34	50	61	52	70
*T* = 4	36	34	47	53	52	65

^*∗*^25th percentile; ^*∗∗*^75th percentile.

**Table 3:** Median, 25th, and 75th percentiles of plasma creatinine (pCrea) and e-GFR in Group B patients with and without AKI diagnosis.

Group B						
	AKI			Non-AKI		
	Median	25th^*∗*^	75th^*∗∗*^	Median	25th^*∗*^	75th^*∗∗*^
pCREA (mg/dL)						
*T* = 0	1.2	1.0	1.4	1.0	0.9	1.3
*T* = 1	1.1	1.0	1.7	1.1	0.9	1.2
*T* = 2	1.3	1.0	2.1	0.9	0.8	1.2
*T* = 3	1.8	2.1	2.9	0.8	0.8	1.2
*T* = 4	2.1	1.5	2.4	0.9	0.8	1.2
e-GFR (mL/min/1.73 m^2^)						
*T* = 0	52	49	54	54	49	83
*T* = 1	49	48	51	54	48	64
*T* = 2	39	26	51	57	50	71
*T* = 3	29	25	42	65	51	76
*T* = 4	21	20	36	69	54	69

^*∗*^25th percentile; ^*∗∗*^75th percentile.
